# Second-Order CASSCF Algorithm with the Cholesky Decomposition of the Two-Electron Integrals

**DOI:** 10.1021/acs.jctc.1c00327

**Published:** 2021-11-01

**Authors:** Tommaso Nottoli, Jürgen Gauss, Filippo Lipparini

**Affiliations:** † Dipartimento di Chimica e Chimica Industriale, Università di Pisa. Via G. Moruzzi 13, I-56124 Pisa, Italy; ‡ Department Chemie, Johannes Gutenberg-Universität Mainz, Duesbergweg 10-14, D-55128 Mainz, Germany

## Abstract

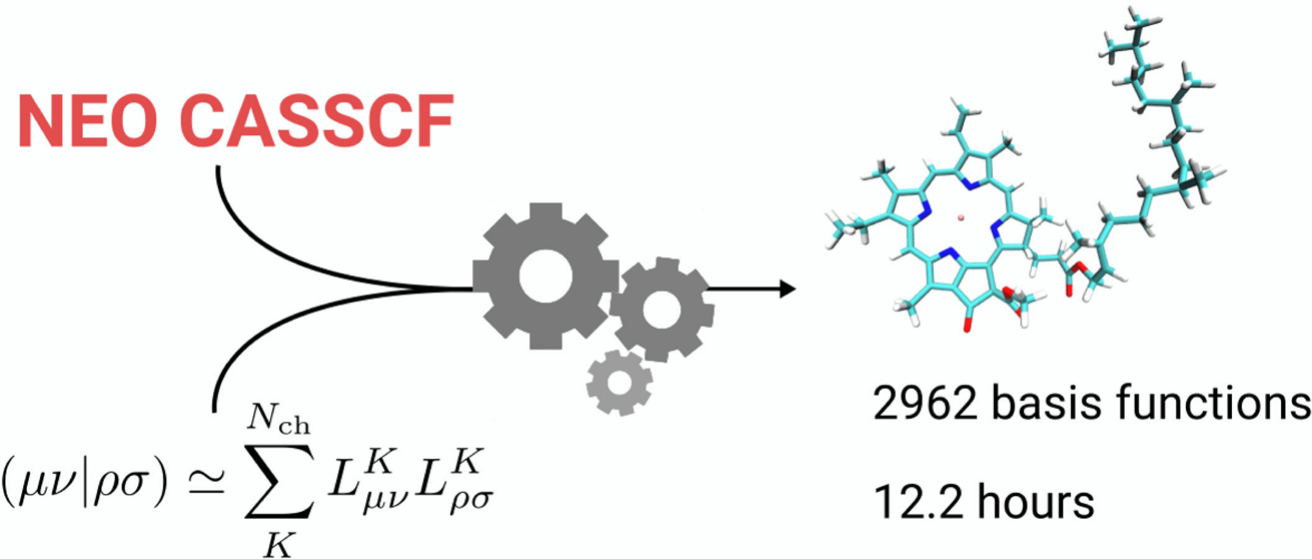

In this contribution, we present the implementation of a second-order complete active space–self-consistent field (CASSCF) algorithm in conjunction with the Cholesky decomposition of the two-electron repulsion integrals. The algorithm, called norm-extended optimization, guarantees convergence of the optimization, but it involves the full Hessian and is therefore computationally expensive. Coupling the second-order procedure with the Cholesky decomposition leads to a significant reduction in the computational cost, reduced memory requirements, and an improved parallel performance. As a result, CASSCF calculations of larger molecular systems become possible as a routine task. The performance of the new implementation is illustrated by means of benchmark calculations on molecules of increasing size, with up to about 3000 basis functions and 14 active orbitals.

## 1 Introduction

The complete active space–self-consistent field (CASSCF) method^1−3^ is a powerful tool to achieve a qualitatively correct description of strongly correlated systems. Thanks to its intrinsic multireference nature, it can be used to compute the structure and molecular properties of a large manifold of interesting systems that are poorly described with standard single-reference methods. These include many open-shell systems, molecules with stretched bonds, and therefore reactivity, excited states, and others. It can also provide a starting point for subsequent high-level correlated treatments, such as internally contracted multireference-configuration interaction^4,5^ (CI) and coupled cluster,^6−13^ multireference perturbation theory such as CASPT2^14,15^ and NEVPT2,^16−18^ or even quantum Monte Carlo methods.^19,20^ Unfortunately, the method suffers from three major complications that restrict its applicability. First, it is not a black box method, as it requires the user to select the active space for the calculation. While there are a few strategies to aid the selection,^21−23^ achieving good results relies still on the user’s chemical intuition and understanding of the system. Second, the CASSCF wave function’s optimization problem is notoriously hard to converge. Third, the method is computationally very demanding.

The computational cost of a CASSCF calculation stems from two concurring factors. The most prominent one is that the method requires one to solve a full CI (FCI) problem in the active space. Due to the combinatorial scaling of FCI, the investigation of large active spaces is not possible using standard direct CI techniques. Approximations to the FCI wave function can be used to overcome this otherwise overwhelming barrier, the most common example being the use of a density-matrix renormalization group^24^ (DMRG). However, many interesting systems can be successfully described with a relatively small active space (up to 12–14 electrons in as many orbitals). If a careful choice of the active space that allows one to capture the static correlation of the wave function with a limited number of active orbitals is possible, the cost of the CI part is either negligible (for active spaces with less than 10 orbitals) or manageable with traditional implementations. In such cases, the cost of the calculation is dominated by the operations involving the manipulation of the electron repulsion integrals (ERIs).

Convergence problems can be mitigated, if not completely solved, by using an optimization algorithm with guaranteed convergence to the closest local minimum. Methods based on a restricted step second-order optimization offer such a guarantee and are, therefore, a very attractive option. However, because they involve the evaluation of the energy Hessian with respect to the variational parameters, i.e., orbital rotations and CI expansion coefficients, they are in general more expensive than their first-order counterparts and require cumbersome and involved implementations. Nevertheless, second-order CASSCF implementations have been successfully achieved and are based on two main algorithms. The first algorithm, originally proposed by Werner and Meyer^25^ and further refined by Werner, Knowles, and others,^26,27^ is based on the definition of a model energy function which is infinite order in orbital rotations and that is optimized. The coupling between CI and orbital optimization is introduced up to second order, ensuring thus quadratic convergence. This algorithm shows excellent convergence properties and overall performances. A similar strategy has been followed by Sun et al.,^28^ and the resulting algorithm, which is based on an integral-direct implementation and can use DMRG as a CASSCF solver, exhibits impressive performances. A second choice is to use a more traditional trust-region second-order method, such as the Levenberg–Marquardt method.^29^ Augmented with an adaptive choice of the trust radius, as proposed by Fletcher (we refer to the global strategy as FLM), it is possible to prove that the FLM method always converges to the closest local minimum and that the rate of convergence is quadratic. A very efficient implementation of the FLM method, known as the norm-extended optimization (NEO) algorithm, has been proposed by Jensen and co-workers.^30,31^ In this contribution, we follow the latter strategy, which we have previously implemented in the CFOUR^32,33^ suite of programs.

A second-order CASSCF implementation requires one to work with ERIs transformed in the molecular orbital (MO) basis with at least two indices spanning the full rank of MOs. The transformation of the ERIs from the atomic orbitals (AOs) to the MO basis is expensive, requiring  floating point operations, where *M* is the number of internal and active orbitals and *N*_b_ the number of basis functions. Furthermore, it is not easily implemented in an efficient way. This is due to the fact that the ERIs matrix is usually too large to fit in memory, especially in the MO basis, which implies that the transformation involves slow disk I/O. Furthermore, the AO ERIs are computed (and stored) in an order that depends on the shell structure of the basis set for the specific system. As a consequence, the integrals are read (or recomputed, in integral-direct implementations) in a system-dependent order, which makes the use of efficient BLAS routines^34,35^ and, more in general, vectorization, particularly challenging.

To address the computational cost involved with the manipulation of the ERIs, it is possible to adopt a low-rank approximation of the ERIs, such as density fitting^36−44^ (DF) or Cholesky decomposition^45−51^ (CD). Both techniques have been successfully applied in many contexts of quantum chemistry,^52−61^ including CASSCF.^62−64^ The CD technique is particularly attractive, as it allows a rigorous, a priori control of the approximation error. Furthermore, it offers a compact representation of the ERIs that is well-suited for vectorized, efficient implementations, as the Cholesky-decomposed ERIs, can be often kept in memory with standard computer hardware and are easily manipulated using highly optimized level 3 BLAS routines. Furthermore, all of the ERIs manipulation can be written as the sum of independent operations on a given Cholesky vector and are therefore very easy to parallelize. Other approaches that aim at large-scale applications are present in literature; see for instance refs (65) and (66), where a first-order implementation on graphical processing units is shown, and the computational cost in the orbital part is mitigated by exploiting the sparsity of the two-electron integrals.

In this contribution, we present an implementation of NEO CASSCF in the CFOUR suite of programs^32,33^ based on the CD of the ERIs. The implementation is tested on several molecular systems of increasing size, for active spaces that go from small (CAS(6,6)) to large (CAS(14,14)) and using up to about 3000 basis functions. This work is organized as follows. In Section 2, the derivation of the NEO CASSCF method is reviewed. The implementation of the algorithm is discussed in Section 3 with a special focus on the Cholesky implementation. In Section 4, benchmark calculations are presented for the purpose of showing the performance of the algorithm in the optimization of medium-to-large systems. Finally, concluding remarks and some perspectives on future developments are given in Section 5.

## 2 Norm-Extended Optimization CASSCF

In this section, we recapitulate the main aspects of NEO CASSCF. First, the parametrization of the wave function is discussed in Section 2.1. Then, the NEO algorithm is briefly summarized in Section 2.2. Further details regarding the optimization algorithm can be found in ref (30) or in a previous work by two of us.^67^

### 2.1 Parametrization of the CASSCF Wave Function

The starting point for the following discussion is given by a set of molecular orbitals (MOs) , where *N*_b_ is the number of basis functions. In CASSCF, the MOs are subdivided into three classes according to their allowed occupation number in a Slater determinant—namely, *internal*, which are always doubly occupied; *active*, which are subjected to no restriction; and *external*, which are always empty. To distinguish an orbital among such classes, the following labels are used: *i*, *j*, *k* refer to inactive, *u*, *v*, *x* to active, *a*, *b*, *c* to external, and *p*, *q*, *r* to generic orbitals. Indices that run over the determinantal space are labeled with capital letters *I*, *J*.

A convenient parametrization for the wave function, first proposed by Jensen and Jørgensen,^30^ is
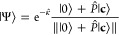
1Here,  is the current approximation to the wave function, or current expansion point (CEP). |**c**⟩ is the correction vector that collects the CI variational parameters, *c*_*I*_,
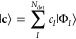
2and *P̂* = 1 – |0⟩⟨0| is the operator that projects |**c**⟩ in the orthogonal complement of |0⟩, thus keeping any redundant vector parallel to the CEP.

Orbital variations are described through a unitary transformation, **φ̅** = **φU**, that is conveniently parametrized by using an exponential map
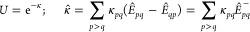
3where  is the spin-traced singlet excitation operator. The variational parameters are given by the elements of the antisymmetric matrix, **κ**. Since only rotations between different orbitals classes produce a variation in the energy, the expression for κ̂ can be simplified as follows:
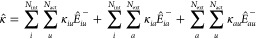
4Hence, **κ** is considered as a vector whose dimension is given by all nonredundant orbitals rotations; i.e., *N*_rot_ = *N*_int_*N*_act_ + *N*_int_*N*_ext_ + *N*_act_*N*_ext_.

### 2.2 Optimization of the CASSCF Wave Function

Equation 1 is used to define a variational expression for the electronic energy that reads
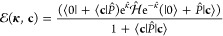
5In eq 5,  is the nonrelativistic Hamiltonian operator written in second quantization
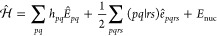
6where
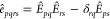
7*h*_*pq*_ are one-electron integrals, (*pq*|*rs*) are two-electron integrals written in Mulliken’s notation, and *E*_nuc_ is the nuclear repulsion term. A second-order algorithm can be developed by defining a quadratic model for the energy; therefore, we expand eq 5 in power series up to second order. To this end, it is useful to define a generic parameter point, **x** = (**c**, **κ**), and the reference one, **x**_0_ = (**c**^(0)^, **0**) such that
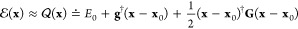
8In eq 8, *E*_0_ is the reference energy, that is , while **g** and **G** are respectively the electronic gradient and Hessian evaluated at the CEP. Analytical expression for such quantities can be obtained by direct differentiation of eq 5 and by exploiting the Baker–Campbell–Hausdorff (BCH) formula. The gradient is given as
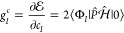
9
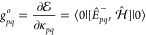
10and the Hessian
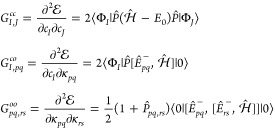
11The minimization of the quadratic model directly leads to the Newton–Raphson (NR) equations. However, the radius of convergence of NR is small, and the Hessian can be non-positive-definite at the beginning of the optimization leading to incorrect search directions. To overcome this issue, a more robust strategy consists of using a trust-region optimization algorithm, e.g., the Levenberg–Marquardt (LM) method,^29^ where the minimization is performed in a reduced domain such that the Hessian has the correct signature. The LM equations can be seen as diagonally shifted NR ones, where the shifting parameter controls the step length to be within a predefined trust radius, *R*_t_. The norm-extended optimization algorithm^30,68^ is an elegant way to recast the LM minimization problem into an eigenvalue–eigenvector one:
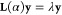
12where **L**(α) is the gradient-scaled augmented Hessian matrix,
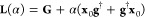
13It can be shown that for ground-state optimization the optimal direction is given by the first eigenvector of **L**(α). Once **y** is given, the NEO step can be computed as
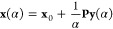
14Here, ***P*** is the matrix representation of the projector operator *P̂*. The step length is controlled by the parameter α and can be obtained by solving the equation
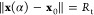
15Eventually, the trust radius is changed adaptively during the optimization procedure according to Fletcher’s algorithm.^29^ If the energy increases, the step is discarded, and the trust radius is decreased. Otherwise, *R*_t_ is either increased or left untouched on the basis of the value of the ratio between the predicted variation of the energy and the actual one. The combined strategy—NEO plus Fletcher’s update—leads to an algorithm that always converges to the closest local minimum for well-behaved wave functions.

## 3 Implementation

In this section, the implementation of the NEO algorithm within the CFOUR^32,33^ suite of programs is discussed. In Section 3.1, we present working expressions for the gradient and for the linear transformations that describe the action of the augmented Hessian matrix on a trial vector. In Section 3.2, the Cholesky decomposition of the two-electron integrals is introduced. Details regarding a cost-effective implementation that exploits the Cholesky vectors are given for a specific example.

### 3.1 Direct NEO Equations

The NEO algorithm can be thought of as a two-level procedure. In the first level—the macroiterations—the parameter hypersurface is scanned by updating the CEP and the MOs. In the second level—the microiterations—a specific NEO eigenvalue–eigenvector problem is iteratively solved with the intention of getting the optimal step direction. At each macroiteration an AOs to MO transformation is performed. Then, the orbital and CI gradient are assembled, and the electronic energy is calculated. The CASSCF energy can be written as
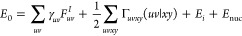
16where γ_*uv*_ and Γ_*uvxy*_ are the one- and two-body reduced density matrices, respectively, which can be computed as the expectation value of the excitation operators
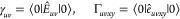
17*F*_*pq*_^*I*^ are the elements of the inactive Fock matrix
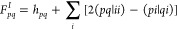
18and *E*_i_ is the energy contribution that stems from the inactive electrons and is called inactive energy,
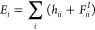
19Manipulation of eq 10 leads to an antisymmetric expression for the orbital gradient
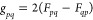
20In eq 20 we have introduced the generalized Fock matrix, whose elements can be written in terms of the inactive Fock matrix, active Fock matrix, and **Q** matrix. The last two are defined as follows:
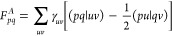
21
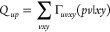
22As eq 9 states, the CI gradient can be evaluated as the action of the Hamiltonian operator on |0⟩
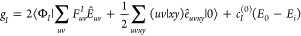
23where the last term is a vector parallel to the CEP that stems from the presence of the projector operator in the wave function definition.

The iterative solution of the NEO eigenvalue–eigenvector problem (microiterations) requires setting up expressions for the matrix–vector product between the augmented Hessian and a trial vector:
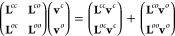
24The present implementation makes use of the split-Davidson algorithm,^68^ where configurations-only, ***v***_conf_ = (***v***^*c*^, **0**), and orbitals-only, ***v***_orb_ = (**0**, ***v***^o^), vectors are added in the Krylov-like subspace. This procedure allows one to adaptively add to the subspace either ***v***_conf_ or ***v***_orb_, depending on the part that exhibits the largest residual. Here we report the expressions for the direct product
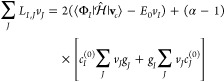
25
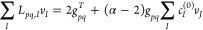
26
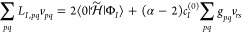
27
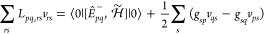
28where |***v***_*c*_⟩ = *∑*_*I*_*v*_*I*_|Φ_*I*_⟩, and  is the one-index transformed Hamiltonian operator. In eq 26, we have introduced the transition gradient *g*_*pq*_^*T*^, that is, a gradient computed with symmetrized transition density matrices. The first term of eq 28 is a gradient-like contribution computed with one-index transformed one- and two-electron integrals. It can be effectively computed by means of the transformed inactive Fock matrix, active Fock matrix, and **Q** matrix whose expressions are given below
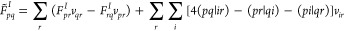
29
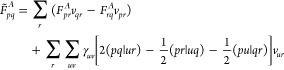
30
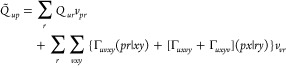
31Explicit expressions for eq 28 are given in the Supporting Information.

**Figure 1:**
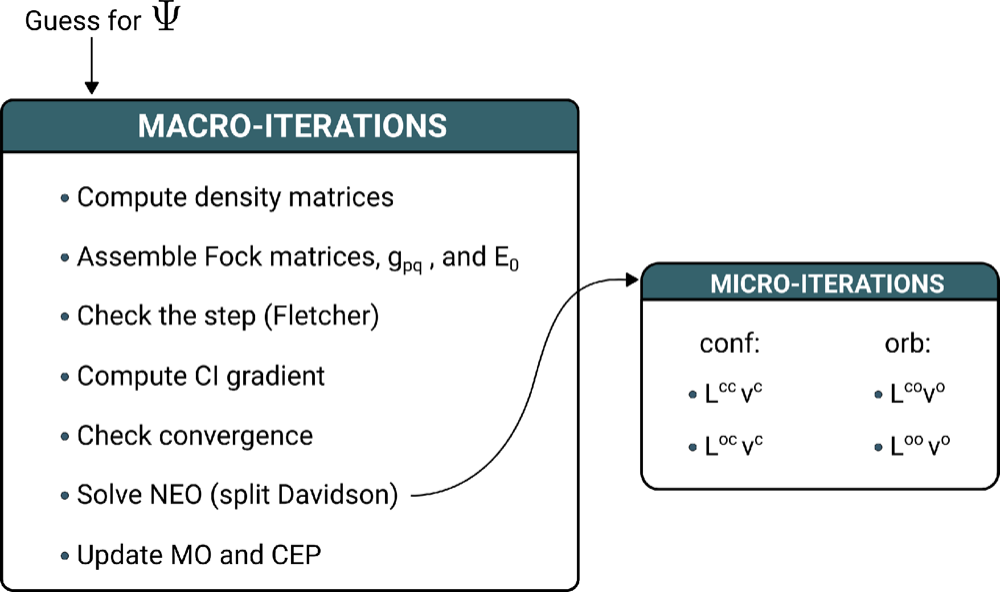
Macro-/microiterations scheme for the NEO algorithm.

The transformed matrices have to be computed at each step of the microiterations, and together with the AO to MO transformation constitute the bottleneck of the algorithm when the chosen active space is small. A summary of the NEO algorithm is given in Figure 1.

### 3.2 NEO Equations with Cholesky Vectors

The ERI matrix is symmetric and positive-semidefinite; therefore, it can be decomposed according to the Cholesky decomposition:
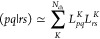
32We compute the CD of the integrals using the partial pivoting algorithm proposed by Koch et al.^47^ (a much more efficient CD algorithm has been recently proposed; see for instance ref (51)), which has been implemented inside the Mainz integral package^69^ (MINT) in CFOUR.^32,33^ The procedure stops whenever the residual of the diagonal is below a user defined threshold. Using the Cauchy–Schwarz inequality, it can be shown that the error on the reconstructed integrals is always lower than or equal to the threshold, so it can be controlled systematically. In eq 32, *N*_ch_ is the number of Cholesky vectors generated; the higher the decomposition threshold the lower the number of Cholesky vectors.

The Cholesky representation of the integrals has been substituted in all equations, namely, the Fock matrices, the transformed Fock matrices, and the active ERI matrix. In order to illustrate the implementation of the evaluation of the aforementioned quantities, we discuss in detail the calculation of the transformed **Q** matrix. Implemented expressions for the transformed Fock matrices can be found in the Supporting Information. Inserting eq 32 into eq 31, we get
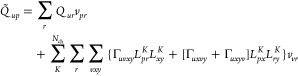
33The first term of eq 33 can be straightforwardly computed from the **Q** matrix. The second term is evaluated by first assembling, for each Cholesky vector, the intermediate quantities
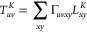
34and
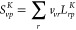
35such that
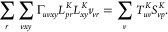
36

Regarding the last term, we notice that  is the fully active part of the intermediate ***S***^*K*^ of eq 35. Hence, we define
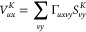
37and
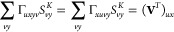
38where in eq 38 we exploited the symmetry of the two-body reduced density matrix. Gathering together eqs 36, 37, and 38, we can rewrite eq 31 as
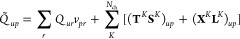
39where *X*_*ux*_^*K*^ = *V*_*ux*_^*K*^ + *V*_*xu*_^*K*^. A detailed discussion of the computational cost and scaling of the present algorithm, together with a critical comparison with other existing CASSCF codes, can be found in Appendix A.

## 4 Benchmarks

In this section we present benchmark calculations to illustrate the performance of the CD-CASSCF implementation. In all of the calculations, convergence is achieved when the root-mean-square norm of both the orbital and CI gradient is below 10^–7^. The threshold for the Cholesky decomposition has been set to 10^–4^.

### 4.1 Importance of a Good Starting Guess

**Figure 2:**
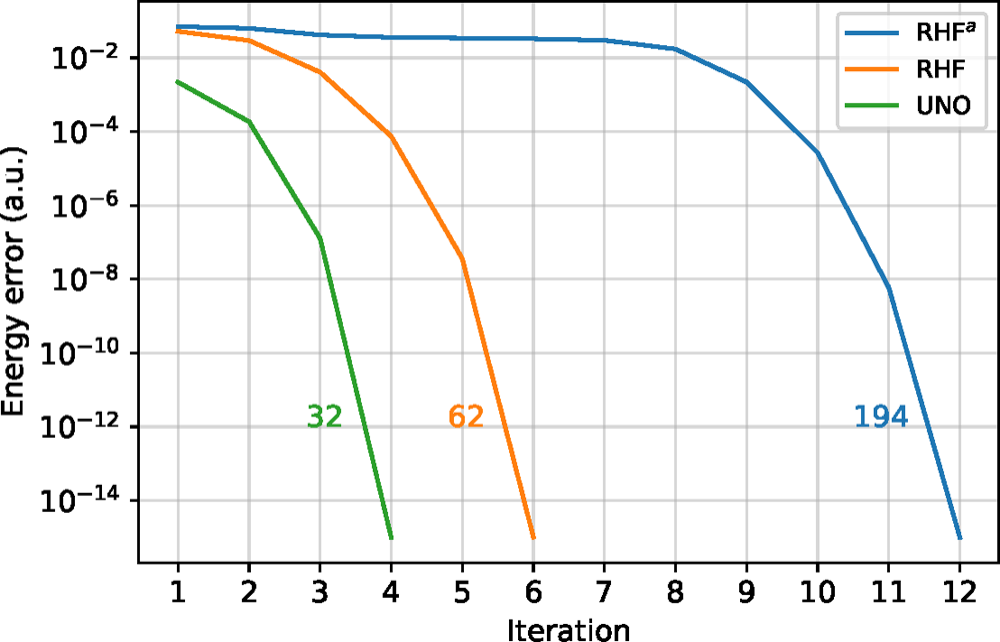
Convergence profile for the CASSCF optimization of the serotonin molecule with 8 electrons in an 8 orbitals active space and 6-31G basis set. In blue (RHF^a^), the active orbitals were selected as the four lowest and four highest canonical RHF; in orange (RHF), as the π/π* canonical RHF; and in green (UNO) as the unrestricted natural orbitals. Next to the three curves, the total number of microiterations is reported. In all three cases the converged CASSCF energy is −569.279 597 357 hartrees.

When using a traditional second-order method, such as the one described in the present work, a good starting guess for the molecular orbitals is beneficial for reducing the number of macroiterations. Also, the quality of the orbitals can affect the number of microiterations required to solve the NEO eigenvalue–eigenvector problem (see eq 24), whose convergence can sometimes be slow. The poor convergence of standard second-order methods has been thoroughly investigated by Werner and co-workers,^25,26^ and it is due to the combination of two facts. As the energy is parametrized in terms of orbital rotations, which are defined as the exponential of a skew-symmetric matrix, a second-order approximation cannot faithfully reproduce the periodicity of rotations. Furthermore, the active space is bound to contain almost doubly occupied and almost empty orbitals. If a HF guess is used, the rotations that mix these orbitals with internal and external ones, respectively, will be associated with very small gradients and small Hessian eigenvalues. This in turn translates into a poor convergence radius of a second-order expansion, which makes the use of a trust-radius algorithm paramount. This motivated Werner, Meyer, and Knowles to use a higher order expansion for their MCSCF algorithm, which allows them to achieve an extremely robust and fast convergence. To better illustrate the importance of a good starting guess when using a traditional second-order algorithm and to further describe the behavior of the optimization procedure when a poor one is used, we analyze the convergence of our implementation using the serotonin molecule as an example and Pople’s 6-31G basis set.^70^ For this example, we use the conventional implementation (without CD). We compare three different starting points. As a production reference, we employ the unrestricted-natural-orbitals (UNOs) criterion of Pulay and Hamilton.^71^ This strategy has been recently cross-validated against other active space selection methods,^23^ and it has been shown to produce almost the same active space as the AVAS-,^22^ FOD-,^72^ and DMRG-based strategies.^21,73^ To compute the UNO, we first look for triplet instabilities in the RHF wave function by computing the lowest eigenvalues of the corresponding instability Hessian.^74^ If one or more negative eigenvalues are present, we perturb the MOs along the direction given by the associated eigenvectors, and we run an UHF calculation. Finally we compute the UNO from the averaged charge density matrix and choose as active orbitals the ones with occupation numbers between 0.01 and 1.99. According to this protocol, the active space for the serotonin consists in 8 electrons and 8 orbitals. A second starting point is obtained from a RHF reference by manually selecting the π/π* orbitals. Lastly, we choose the four highest occupied and four lowest unoccupied canonical RHF orbitals. In Figure 2, we report the convergence profile (energy difference with respect to the converged solution as a function of the number of macroiterations) for the three aforementioned starting points. We also report the overall number of microiterations (summed over the number of macroiterations).

**Figure 3:**
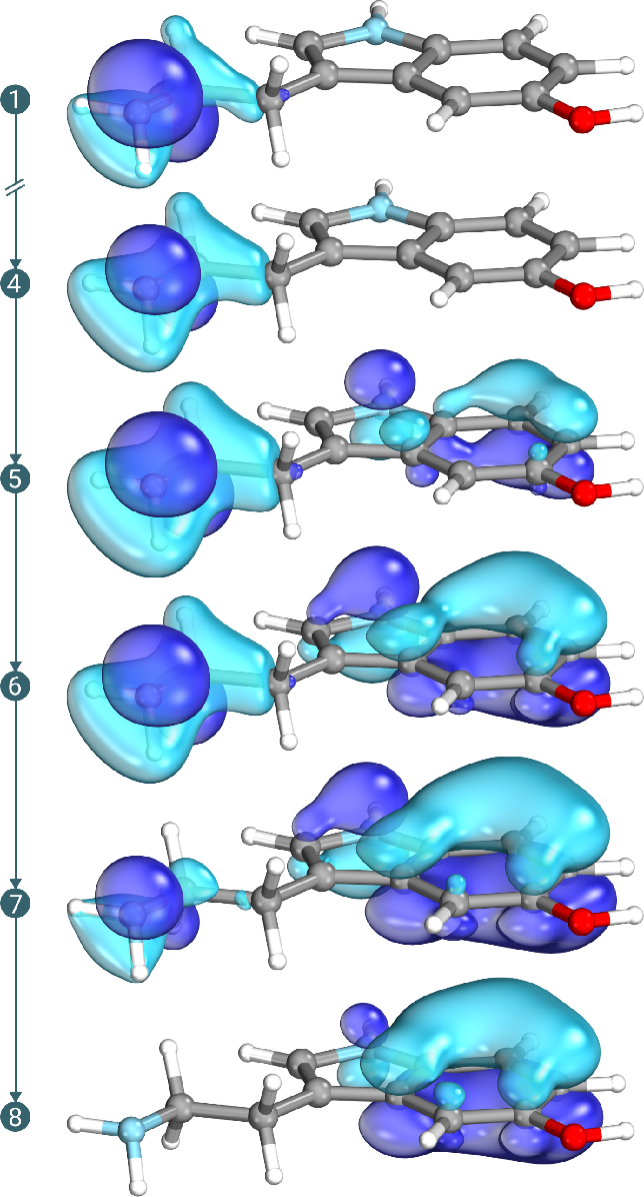
Snapshots of the lowest energy active orbital taken at various CASSCF macroiterations. Reported are only iterations where a qualitative change on the orbital’s shape is visible. The starting guess for the orbitals was given by the canonical RHF ones, without any manual choice. The orbitals were visualized using the IboView software.^75,76^

All three calculations converged to the same state. The calculation using the RHF π/π* orbitals converged relatively quickly, but the presence of small eigenvalues in the MO rotation at the beginning of the calculations requires two additional iterations with respect to the calculation starting from the UNO, during which small steps are taken and the gradient remains relatively unchanged. As expected, the last choice presents the worst convergence trend, with the first eight iterations being spent looking for the quadratic region. This can be easily understood. To achieve convergence, the orbitals have to be swapped, which corresponds to a large orbital rotation. Due to the small convergence radius of a second-order expansion, this requires in turn a large number of steps. We illustrate this fact in Figure 3, where we represented the lowest energy MO at various steps of the optimization (the corresponding iterate is reported to the left of the MO picture). We note that in the first steps the orbital changes its shape—the rotation with a π inactive orbital is gradually magnified during the optimization.

This example shows that a good starting guess and a careful selection of the active orbitals are paramount to achieve a smooth and fast convergence. Nevertheless, it also shows that the NEO algorithm is robust and is able to achieve convergence even when starting from a particularly bad reference.

### 4.2 Benchmark Calculations

**Figure 4:**
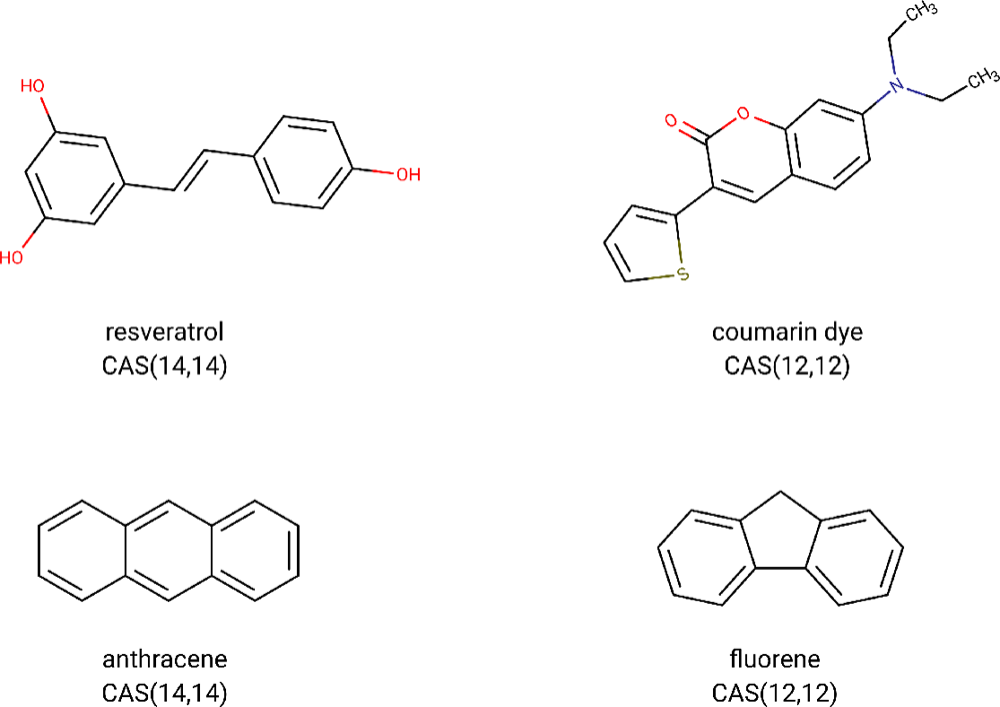
New aromatic molecules used to test the CD-CASSCF algorithm together with their active spaces.

**Figure 5:**
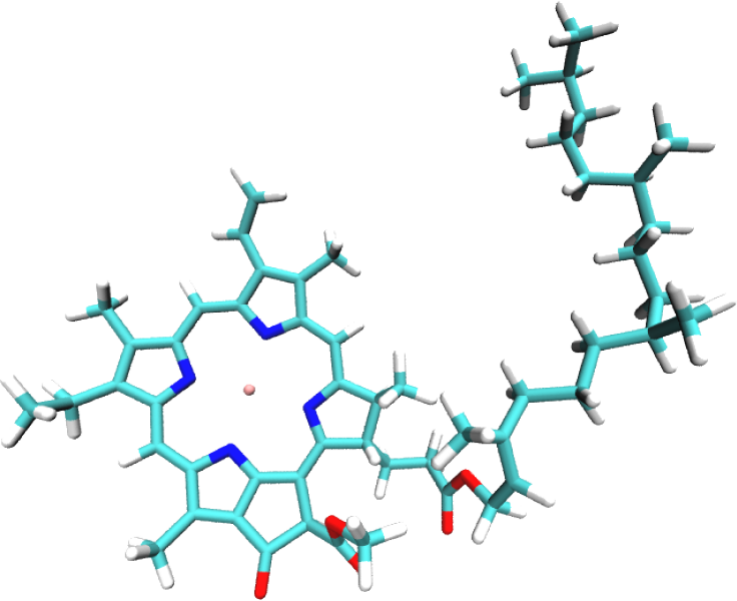
Structure of the chlorophyll molecule.

The starting benchmark set used to test the CD-CASSCF code is composed of 21 aromatic molecules. The geometries were taken from ref (77); the set was used also by Kreplin et al. to test their MCSCF solver.^27,78^ We augmented the benchmark set with 5 larger molecules whose geometries were optimized at the B3LYP/6-31G(d)^70,79^ level of theory using the Gaussian 16 suite of programs.^80^ All of the new structures can be found in the Supporting Information; a pictorial representation of the molecules is given in Figures 4 and 5. All calculations were done using Dunning’s cc-pVTZ^81^ basis set using spherical harmonics.

As a starting analysis, we compared the storage requirement of a CD-based CASSCF calculation with a conventional one. The exact CD of the ERI matrix would generate *N*_b_(*N*_b_ + 1)/2 Cholesky vectors. We define a compression rate
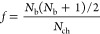
40to measure the effectiveness of the truncated CD in reducing the dimension of the computational problem. We report in Table 1, for seven selected molecules from the benchmark set, the number of basis functions (*N*_b_), the disk space needed to store the two-electron integrals, and the compression rate.

**Table 1:** Systems Used to Compare the Different Storage Requirement of the Standard and CD Implementations in CFOUR^a^

			size (GB)	
molecule	*N*_*b*_	*f*	CD	STD
catechol	324	24.88	0.9	18.3
naphthalene	412	31.98	1.8	43.1
nicotine	556	43.46	4.4	139.7
tryptophan	618	47.75	6.1	170.6
pyridoxamine	528	41.00	3.8	108.8
2Me4HSdiox	446	34.10	2.3	61.8
indole	368	28.37	1.3	30.4

a For each molecule, we report the number of basis functions (*N*_*b*_), the compression factor (*f*) defined in eq 40, and the size in gigabytes of the Cholesky vectors (CDs) and two-electron integrals (STD).

**Figure 6:**
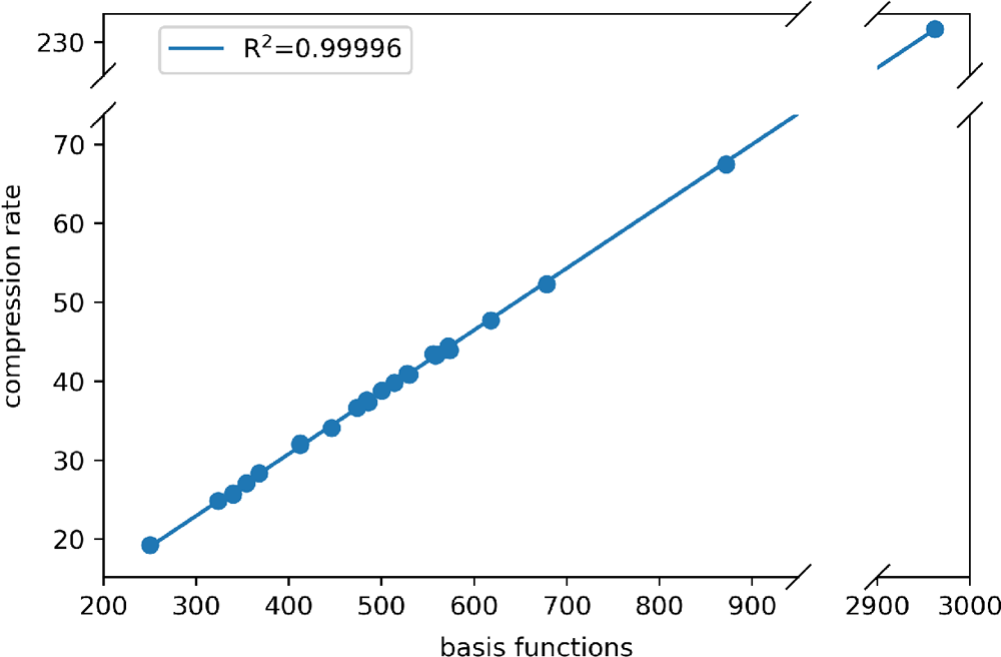
Compression rate trend with respect to the number of basis functions. The points are fitted using standard linear regression.

As expected, the storage requirements for the CD vectors are significantly lower than for the standard two-electron integrals. It is worth remarking that, even for the largest system of this reduced set, the Cholesky vectors can easily be kept in memory even on a standard desktop computer. This is one of the main advantages of using a reduced order approximation of the ERIs, as it allows one to perform full in-core calculations, avoiding thus slow disk I/O operations. In Figure 6, we plotted the compression rate for the whole benchmark set with respect to the number of basis functions. According to our results, we deduce a linear scaling of *f* with respect to the system’s size. Therefore, we can obtain an even more compact representation of the integrals and, thus, a greater efficiency of the CD, with larger systems.

The previously selected molecules were also used to compare the accuracy of the CD-CASSCF energy with respect to the one obtained with the standard algorithm. The chosen decomposition threshold (10^–4^) allows us to obtain a high compression rate while retaining an overall good accuracy. In Table 2, we report the active space (CAS) and the converged CASSCF energy obtained with both the Cholesky and standard implementations for the selected molecules. As it can be seen from the table, the two results are in agreement to at least the fourth decimal digit, with the largest deviation being about 50 μ*E*_h_. We note that it has been documented in the literature that the errors in CD energies are systematic such that CD benefits from error cancellation, thus further increasing the accuracy of energy differences.^62,82^

**Table 2:** Comparison between the Converged CASSCF Energy of the Cholesky and Standard Implementation^a^

molecule	CAS	CD energy	STD energy
catechol	6,6	–380.624 556 05	–380.624 560 70
naphthalene	10,10	–383.592 108 03	–383.592 112 05
nicotine	6,6	–495.948 509 96	–495.948 509 88
tryptophan	8,8	–682.501 156 65	–682.501 137 25
pyridoxamine	6,6	–568.806 086 69	–568.806 101 53
2Me4HSdiox	6,6	–855.034 927 82	–855.034 975 73
indole	8,8	–361.673 841 72	–361.673 848 10

a Energy values are given in hartrees.

To test the performances of the CD-CASSCF algorithm, we run calculations on the whole benchmark set that involved active spaces up to CAS(14,14) and as many as 2962 basis functions. For each system, all of the orbitals, including the core ones, are fully variationally optimized. All of the calculations presented here were performed on a single cluster node equipped with 4 Intel Xeon Gold 6140 M CPUs, running at 2.30 GHz. The lower triangular part of the Cholesky vectors were kept in memory. Shared-memory parallelization is exploited in all of the calculations, sharing the work among 28 cores. We point out here that we do not expect the implementation to be fully scalable, the limiting factor being the full CI code. This is due to the fact that the sequential code is highly cache-optimized, which causes an overload of the cache, and consequent loss of efficiency, when more cores of the same processor share cache access. Nevertheless, even a straightforward OpenMP parallelization of the main loops is beneficial. In Table 3 we report, for each molecule, the active space (CAS), the number of basis functions, the number of macroiterations required to converge, and the total CPU wall time in minutes.

**Table 3:** Benchmarks Set Results^a^

molecule	CAS	*N*_*b*_	it.	time (min)
adrenaline	6,6	572	4	1.04
anthracene	14,14	560	5	8.27
azulene	10,10	412	5	0.57
biphenyl	12,12	500	4	0.93
catechol	6,6	324	4	0.11
dopamine	6,6	484	4	0.63
fluorene	12,12	530	4	0.95
indole	8,8	368	5	0.31
l-dopamine	6,6	574	4	1.08
naphthalene	10,10	412	4	0.34
niacin	6,6	340	4	0.14
niacinamide	6,6	354	4	0.17
nicotine	6,6	556	4	0.85
nor-adrenaline	6,6	514	4	0.74
picolinic acid	6,6	340	4	0.16
pyridine	6,6	250	4	0.05
pyridoxal	8,8	486	5	0.88
pyridoxamine	6,6	528	5	1.21
pyridoxin	6,6	514	4	0.86
resveratrol	14,14	678	5	11.43
serotonin	8,8	558	4	0.95
tryptophan	8,8	618	5	2.08
2Me2HSdiox	4,4	474	5	0.76
2Me4HSdiox	6,6	446	5	0.61
coumarin dye	12,12	872	5	4.55
chlorophyll	12,12	2962	12	735.01

a For each molecule, the active space (CAS), the number of basis functions (*N*_*b*_), the number of macroiterations (it.), and the total CPU wall time (time) in minutes are presented. 2Me2HSdiox is the abbreviation for 5,7-dimethyl-2*H*,3*H*-thieno[3,4-*b*][1,4]dioxine.

For all of the systems, we used as MO guess the UNO. If more than one instability is found, we compute the UNO from the averaged one-body charge density matrix of the various unrestricted solutions, the only exception being the chlorophyll molecule where we compute the UNO following a single instability (out of three)—the one with the highest negative eigenvalue in absolute value. This choice is in principle suboptimal, but unfortunately, when we performed the computation with a smaller basis set (cc-pVDZ), we found that the optimal active space prescribed by the UNO strategy, a CAS(22,22), was out of the reach of a traditional full CI implementation. To obtain a feasible active space, we reduced the occupation thresholds used to select the active orbitals from 0.01–1.99 to 0.05–1.95, which resulted in a CAS(12,12). We verified that, with this choice, the cc-pVDZ calculation with the UNO obtained following a single instability converged to the same minimum and exhibited the same convergence pattern as the one using the UNO obtained following all of the instabilities.

All of the calculations for which the full active space built using the UNO procedure was computationally affordable, i.e., all but the one on chlorophyll, converged in at most 5 iterations, which demonstrates not only the robustness of the NEO algorithm and the overall efficiency of the implementation but also the remarkable quality of the UNO guess. Unfortunately, the active space suggested by the procedure for chlorophyll was out of reach of a traditional full CI solver. As a consequence, we had to reduce it to a more manageable CAS(12,12). The non-optimality of such a choice is reflected in the larger number of iterations required to converge the wave function. Nevertheless, the overall calculation could be completed in little more than 12 h.

To further illustrate the behavior of the algorithm, we can divide the work into three main tasks—the AO to MO transformation, the optimization of the MOs (MO opt.), and the optimization of the CI coefficients (CI opt.). The MO optimization includes the calculation of the orbital gradient (eq 20), which in turn requires one to assemble the various Fock matrices (eqs 18, 21, and 22), the calculation of the diagonal of the MO Hessian (which is used as the preconditioner in the Davidson diagonalization), and the evaluation of the direct eqs 27 and 28 for each microiterations. On the other hand, the CI optimization consists of computing the reduced density matrices, assembling the CI gradient (eq 23), and evaluating eqs 26 and 25 at each microiteration. Table 4 shows the percentage time to perform these three operations with respect to the total time of the last macroiteration, which is usually the one that requires the largest number of microiterations to converge the NEO problem (microit. in Table 4).

**Table 4:** Percentage Time of the Three Leading Operations with Respect to the Total Time of the Last Macroiteration (Time)^a^

molecule	AO to MO (%)	MO opt. (%)	CI opt. (%)	microit.	time (s)
adrenaline	8.76	90.63	0.04	20	29.0
biphenyl	5.06	71.07	22.0	12	21.4
naphthalene	7.83	82.41	7.67	13	6.6
tryptophan	5.75	93.54	0.14	24	42.2
anthracene	0.98	14.35	81.65	11	194.9

a AO to MO refers to the atomic orbitals to molecular orbitals transformation of the Cholesky vectors, MO opt. is the time spent in the MO optimization and includes operations such as calculation of the orbital gradient, evaluation of the NEO augmented Hessian–orbital trial vector products. CI opt. refers to the CI optimization and include the following operations: calculation of the CI gradient, calculation of the reduced density matrices, and evaluation of the NEO augmented Hessian-configuration trial vector products. microit. is the number of microiterations required to solve the NEO eigenvalue–eigenvector problem.

The CD extremely facilitates the integrals transformation, shifting the bottleneck to the MO optimization part. For the systems considered, in particular, most of the time is spent in computing the transformed Fock matrices, an operation that is required to assemble the NEO Hessian-orbital trial vector product (eqs 27, 28). We also note that, for larger active spaces, such as in biphenyl, the cost associated with the CI part starts to become non-negligible and rapidly becomes the bottleneck as shown for anthracene. Here, the most expensive operations are the direct-CI steps needed to compute the CI gradient and the CI part of the NEO augmented Hessian-configuration trial vector products, together with the assembling of the reduced density matrices. As it can be seen, these operations take about 80% of the total time.

## 5 Conclusions

We have presented the implementation of a second-order CASSCF optimization algorithm that exploits the Cholesky decomposition of the two-electron integrals. The algorithm is based on a trust-region method, which requires one to solve diagonally shifted Newton–Raphson equations known as Levenberg–Marquard equations. Also, it adaptively modifies the trust radius during the optimization according to the value of the energy with the result that the overall algorithm always converges to the closest minimum for regular enough functions. The coupling between orbitals and CI coefficients is naturally included in the off-diagonal blocks of the Hessian matrix, making this algorithm naturally second order in all parameters. The implementation is based on the norm-extended optimization formalism, where the LM equations are recast into an eigenvalue problem, where the first eigenvector provides the optimal direction for ground-state minimization problems.

To reduce the computational cost associated with orbital optimization, which is dominating for not-too-large active spaces, we implemented the NEO algorithm using the Cholesky decomposition of the two-electron integrals matrix. The NEO equations were rewritten in terms of the Cholesky vectors, taking particular care in recasting all of the equations in a way that allowed us to implement them efficiently thanks to an extensive use of level 3 BLAS routines. The implementation exploits a fully direct algorithm where the Hessian matrix is never explicitly calculated. Furthermore, since the Cholesky vectors are independent among the others, the code has been parallelized with shared-memory OpenMP directives.

The resulting algorithm was tested on various aromatic systems. We used a triple-ζ basis set with up to 2962 functions and active spaces up to CAS(14,14). All calculations converged swiftly and required limited computer time. Thanks to the effective compression of the two-electron integrals matrix operated by the CD, fully in-core calculations are possible for most systems, eliminating thus the bottleneck of slow disk I/O. While several further improvements and optimizations are possible, for instance, to improve the convergence of the microiterations, the benchmark calculations reported in this contribution show that a rigorous second-order algorithm can be used in large-scale applications at a reasonable computational cost. Future work will focus on both algorithmic improvements and extensions of the methodology. In particular, a first-order procedure such as super CI^83,84^ could be used in the preliminary phase of a calculation to achieve an initial intermediate convergence goal, thus providing a very good starting point for the quadratically convergent optimization. We also plan to extend the second-order procedure to the simultaneous optimization of several electronic states and to the calculation of analytical gradients, by implementing differentiated Cholesky vectors.^63,85^

## Appendix A: Computational Cost and Scaling of the CD-NEO Strategy and Comparison with Different Implementations

In this appendix, we discuss in detail the computational cost of the various operations required to perform a NEO-CASSCF calculation using the CD of the two-electron integrals (CD-NEO) and briefly compare it to other CASSCF optimization strategies. We focus the discussion on the cost of orbital-related operations and therefore neglect the cost of the various direct CI steps. The latter become dominant for large active spaces and are of little importance for small ones.

Second-order methods require two-electron integrals in the MO basis with up to two virtual indices. This requires, in general, to perform  floating point operations and is the main bottleneck in CASSCF calculations not dominated by the CI part. In our implementation, the MO transformed integrals are obtained by fully transforming each Cholesky vector to the MO basis, at a cost of . This is formally still the rate-determining step in our algorithm; however, it can be performed very efficiently using level 3 BLAS routines and is trivially parallelized. Note that we never assemble the two-electron integrals explicitly, except for the ones with four active indices that are needed for the CI problem. The evaluation of the orbital gradients requires one to assemble the inactive and active Fock matrices and the **Q** matrix. Reporting only the leading terms, this requires one to perform , , and  floating point operations, respectively. Again, all of these operations can be performed using level 2 and level 3 BLAS routines and are easily parallelized. All of the operations described so far need to be performed at each macroiteration. Assembling the NEO step requires the solution to the NEO eigenvalue (eq 12). This is done using an iterative procedure and, in particular, Davidson diagonalization. The calculation of the required matrix-vector products requires one to assemble one-index transformed Fock and **Q** matrices, as described in section 3. The leading terms in computational cost for such operations are  and , and therefore the computation of a matrix–vector product is cheaper than the transformation of the two-electron integrals. Once again, the various operations can be performed using efficient BLAS routines and are easily parallelized.

The memory requirements of the CD-NEO strategy are in principle very limited, with a few  arrays being required to assemble various intermediates—it should be noted that such intermediates are duplicated for parallel execution, therefore increasing the memory requirements of the run proportionally to the number of cores used for the calculation. However, the overall algorithm achieves significant speed ups if the Cholesky vectors can be held in core, which requires *N*_ch_*N*_b_(*N*_b_ + 1)/2 words of memory. If such an amount of memory is not available, the Cholesky vectors can be read in batches and then distributed among the available cores for computation. This, however, introduces a significant I/O bottleneck that can not only slow the calculation but also reduce the parallelization efficiency, even though modern hardware equipped with solid-state disks mitigates the loss of performance. Nevertheless, calculations on medium- to large-sized molecules can be performed usually by holding the Cholesky vectors in core. The biggest calculation reported in section 4, where about 3000 basis functions were used, required about 600GB of memory, which is a large, yet manageable, amount of RAM. Calculations for smaller systems, with up to 1000 basis functions, can be easily performed in core even on modest hardware.

It is interesting to compare here the computational cost of the CD-NEO strategy with a different second-order algorithm, the Werner–Meyer–Knowles (WMK) algorithm,^25,26^ as it is one of the most well-established strategies to solve the CASSCF optimization problem. In particular, a very efficient implementation of the WMK exists that relies on density fitting as a compression technique to handle the two-electron integrals.

CD and DF are intimately related from a theoretical point of view, to the point that the Cholesky decomposition can be considered a specific case of DF, where the auxiliary basis, here called the Cholesky basis, is automatically generated by the decomposition algorithm. In DF, the AO product densities are expanded in terms of optimized basis functions; therefore, the two-electron integrals are approximated as

41

where *K* and *J* are elements of the fitting basis and (*K*|*J*) is the overlap matrix of such functions, i.e., the metric. Different metrics can be used, where one considers either the straightforward overlap of the two functions or their Coulomb interaction, the latter being the preferred choice as it can be proven that it affords an approximation of the integrals that is correct up to first order.^86^ According to eq 41, the Cholesky vectors can be expressed as

42

with *B*_*KJ*_ being the Cholesky factor of the matrix (*K*|*J*). When using an orthonormal Cholesky basis (*B*_*KJ*_ = δ_*KJ*_), the Cholesky vectors are exactly the fitting coefficients. Therefore, the CD is a DF procedure with a particular auxiliary basis.

From a computational point of view, there are, however, several differences. DF uses optimized, precomputed basis sets and requires thus only the computation of the inverse metric. The latter operation is in principle expensive if direct linear algebra techniques are used, but the inversion can be obtained at a reduced cost by using local approximations.^44,87^ On the other hand, Cholesky vectors have to be determined for each system and each geometry. This is in practice not a major source of overhead, as very efficient algorithms have been recently proposed.^51^ It is also important to remark that CD allows for easy and efficient parallelization of the code, as all of the operations involving different Cholesky vectors can be performed independently. As a consequence, parallelization is easily achieved by distributing the Cholesky vectors among the available processors, with a final reduction to be performed on the computed quantities. In our implementation, we exploit this feature by implementing all of the various operations with the loop over the Cholesky vector as the most external one. Even a simple-minded shared-memory parallelization with OpenMP directives^88^ is already quite effective. Due to the theoretical similarities between CD and DF, the scaling of the various steps needed to perform a DF-WMK calculation are equivalent to the ones observed in a CD-NEO one. In particular, the leading computational step in DF-WMK is again the transformation of the ERI into the MO basis, for which the authors^78^ report a cost of , *M* being the number of auxiliary basis functions, which is comparable with the  one for CD-NEO. The same applies to the calculation of the MO gradient and to the optimization of the WMK model function (i.e., the WMK microiterations), which require one-index transformed quantities similar to the ones reported here. While a thorough comparison of the timings associated with the two implementations would be beyond the scope of this work, preliminary tests performed on the system used as a benchmark by Kreplin et al.^78^ show very similar performances. Comparing the results reported in the literature for systems of similar size as the ones reported in section 4, we also observed overall similar timings. We stress here that this is only a qualitative comparison and that a more thorough comparison is needed to precisely assess the relative performances of the two implementations.

As a general remark, because the Cholesky vectors are precomputed, we expect a CD-based strategy to be particularly attractive in a pre-asymptotic regime, i.e., when the sparsity of the two-electron integrals matrix is not too pronounced. This includes calculations on compact molecules but also calculations that use large basis sets, especially if diffuse functions are included. For even larger systems, there are two factors that make a CD-based approach less competitive. First, the Cholesky vectors may not fit in memory, requiring thus slow disk I/O. Second, the sparsity of the two-electron integrals is poorly exploited with CD. On the contrary, DF-based approaches have the advantage of being operable using the conventional integral-direct techniques, which allow one not only to compute efficiently the required fitted integrals, but also to achieve reduced (up to linear) scaling regimes when assembling the Fock and Fock-like matrices thanks to the use of advanced integral evaluation methods, such as the J-engine,^89^ continuous fast multipole method,^90^ and various screening techniques^44,87,91^ that efficiently exploit the sparsity in the integrals. Therefore, for very large systems, DF techniques are expected to achieve more significant speed ups than the CD. It should be stressed, however, that computational efficiency is not the only criterion of interest when comparing two different approaches. While the accuracy of the DF approach depends on the reliability of the fitting basis, which needs to be optimized for both the quantum chemistry model and the basis set, the accuracy of the CD is solely controlled by the decomposition threshold, making this approach virtually ab initio and model independent—we can continuously move from an approximate to a near exact description of the integrals. This feature is, in our opinion, particularly attractive, especially for applications for which the numerical accuracy of the results is fundamental.

It is also interesting to compare second-order strategies to first-order ones, where the MOs and CI coefficients are optimized separately in an alternating gradient spirit. First-order methods require MO integrals with up to one virtual index, and therefore the integral transformation costs , which can be reduced to  using CD or, analogously, DF. In the pre-asymptotic regime, this is the leading contribution to the overall cost of such methods for what concerns orbital optimization, making such methods particularly efficient and well-suited for large-scale calculations. Furthermore, first-order methods are well-suited for being implemented in an integral-direct fashion, assembling the various Fock matrices needed to compute the MO gradient in the AO basis. This allows one to fully exploit the sparsity of the ERI matrix in the asymptotic regime, achieving an overall scaling that is between  and , as reported by Hohenstein et al.^65^ in a recent paper. Furthermore, in such an implementation the AO integrals and their contraction with density matrices are performed using GPUs, achieving impressive performances. For very large molecules, especially when compact basis sets are used, this strategy is extremely efficient and bound to overperform any second-order implementation. While a thorough comparison of such an implementation with our CD-NEO strategy is out of the scope of this work, preliminary tests show comparable performances for medium–large systems, that is, as long as the Cholesky vectors fit in memory and the sparsity of the ERI matrix is not too prominent. On the other hand, first-order methods have no guarantee of convergence and can require a very large number of iterations. The robustness of second-order procedures is, in our opinion, a very important feature, especially when one considers the notorious difficulty of converging a CASSCF calculation. Note that, in the pre-asymptotic regime, first-order methods can greatly benefit from the use of CD.^62^ In conclusion, the CD-NEO strategy represents a very good compromise between robustness and efficiency for not-too-large systems, as it was demonstrated in section 4.

## Supporting Information Available

Explicit equations for the direct product with the MO Hessian, implementable expressions for the transformed Fock matrices, and optimized molecular geometries for the new set of aromatic molecules used as benchmarks (PDF)
