# Supplementary material for: Second-Order CASSCF Algorithm with the Cholesky Decomposition of the Two-Electron Integrals
Source: J Chem Theory Comput. 2021 Nov 1;17(11):6819–31. doi: 10.1021/acs.jctc.1c00327 (PMC8582256; doi:10.1021/acs.jctc.1c00327)
Supplement: Supplementary file 1 — ct1c00327_si_001.pdf [file ct1c00327_si_001.pdf]

Supporting Information: A Second-Order CASSCF Algorithm with the Cholesky Decomposition of the Two-Electron Integrals

Tommaso Nottoli,† Jürgen Gauss,‡ and Filippo Lipparini*,†

†*Dipartimento di Chimica e Chimica Industriale, Università di Pisa. Via G. Moruzzi 13, I-56124 Pisa, Italy*

‡*Department Chemie, Johannes Gutenberg-Universität Mainz, Duesbergweg 10-14, D-55128 Mainz, Germany*

E-mail: filippo.lipparini@unipi.it

S1 Direct product with the MO Hessian

In the following, the expressions for the direct matrix-vector product $\sigma_{pq} = \sum_{rs} L_{pq,rs} v_{rs}$

$$\begin{aligned}\sigma_{ix} = 4(\tilde{F}^I_{ix} + \tilde{F}^A_{ix}) - 2\left[\sum_u \gamma_{xu}\tilde{F}^I_{iu} + \tilde{Q}_{xi}\right] + \\ -\sum_y (F_{xy} - F_{yx})v_{iy} + \sum_a F_{xa}v_{ai} + 2\sum_a (F^I_{ia} + F^A_{ia})v_{xa},\end{aligned} \tag{S1}$$

$$\sigma_{ja} = 4(\tilde{F}^I_{ja} + \tilde{F}^A_{ja}) + \sum_x F_{xa}v_{jx} + 2\sum_x (F^I_{jx} + F^A_{jx})v_{ax} - \sum_x F_{xj}v_{ax}, \tag{S2}$$

$$
\begin{aligned}
\sigma_{ya} = 2\left[\sum_u \gamma_{yu}\tilde{F}^I_{au} + \tilde{Q}_{ya}\right] - 2\sum_i (F^I_{ai} + F^A_{ai})v_{iy} + 2\sum_j (F^I_{yj} + F^A_{yj})v_{ja} - \\
+ \sum_j F_{yj} v_{aj} + \sum_x (F_{yx} - F_{xy})v_{ax}.
\end{aligned}
\tag{S3}
$$

S2 Transformed Fock matrices

Here we are not showing the terms that do not involve ERIs elements, since they are given by simple matrix-matrix multiplications that do not require the definition of intermediate quantities. Implementable expression for the transformed inactive Fock matrix:

$$
\tilde{F}^I_{pq} = 4\sum_K f^K L^K_{pq} - \sum_K (\boldsymbol{T}^K \boldsymbol{L}^K)_{pq} - \sum_K ((\boldsymbol{T}^K \boldsymbol{L}^K)^T)_{pq} \tag{S4}
$$

where we have defined the intermediates

$$
f^K = \sum_{ir} L^K_{ri} v_{ri}, \tag{S5}
$$

$$
T^K_{pi} = \sum_r L^K_{pr} v_{ri}, \tag{S6}
$$

$$
(\boldsymbol{T}^K \boldsymbol{L}^K)_{pq} = \sum_i T^K_{pi} L^K_{iq}. \tag{S7}
$$

Implementable expression for the transformed active Fock matrix:

$$
\tilde{F}^A_{pq} = 2\sum_K d^K L^K_{pq} - \frac{1}{2}\sum_K (\boldsymbol{U}^K \boldsymbol{L}^K)_{pq} - \frac{1}{2}\sum_K ((\boldsymbol{U}^K \boldsymbol{L}^K)^T)_{pq} \tag{S8}
$$

where

$$R^K_{pu} = \sum_r L^K_{pr} v_{ru}, \tag{S9}$$

$$d^K = \sum_{uv} \gamma_{uv} R^K_{uv}, \tag{S10}$$

$$U^K_{pu} = \sum_v R^K_{pv} \gamma_{vu}, \tag{S11}$$

$$(\boldsymbol{U}^K \boldsymbol{L}^K)_{pq} = \sum_u U^K_{pu} L^K_{uq}. \tag{S12}$$

S3 New benchmark set geometries

Below, the molecular geometries (in Ångström) optimized at B3LYP/6-31G*.

```
coumarin dye
C       2.882143   -0.268210   -0.309622
C       1.972271    0.816116   -0.260979
C       0.608758    0.571338   -0.188748
C       0.057337   -0.725794   -0.150825
C       0.972634   -1.802914   -0.186738
C       2.337324   -1.591194   -0.258852
H       2.294170    1.846506   -0.272913
C      -1.358769   -0.860468   -0.072090
H       0.590102   -2.818659   -0.152951
H       2.992892   -2.450383   -0.273264
C      -2.218826    0.216286   -0.032646
C      -1.654127    1.572720   -0.070758
H      -1.766510   -1.866461   -0.042927
O      -0.236934    1.670240   -0.147549
O      -2.265635    2.643267   -0.043427
N       4.248837   -0.056609   -0.417262
C       5.199212   -1.183422   -0.440179
H       6.115364   -0.819918   -0.916515
H       4.807416   -1.969877   -1.096012
C       4.802631    1.309817   -0.456008
H       4.178698    1.926779   -1.113240
H       5.783430    1.245802   -0.938163
C       4.947671    1.980508    0.921090
H       5.645551    1.425927    1.556911
H       5.330221    3.001472    0.805325
H       3.986266    2.034248    1.441303
C       5.535406   -1.764948    0.944143
H       6.223001   -2.613028    0.841871
H       6.014481   -1.013300    1.579774
H       4.634497   -2.115192    1.458000
C      -3.665668    0.070204    0.046002
C      -4.655365    1.025096    0.096090
S      -4.417028   -1.603675    0.092450
C      -5.987651    0.500669    0.170022
```

H	-4.421534	2.079256	0.079541
C	-6.053895	-0.861130	0.178437
H	-6.866579	1.132339	0.215329
H	-6.916838	-1.506548	0.227349

fluorene

C	0.000000	0.000000	1.832720
C	0.000000	1.184661	0.886696
C	0.000000	0.734880	-0.450318
C	-0.000000	-0.734880	-0.450318
C	-0.000000	-1.184661	0.886696
C	0.000000	2.545382	1.172195
C	0.000000	3.461910	0.114800
C	0.000000	3.016477	-1.211823
C	0.000000	1.651282	-1.504447
C	-0.000000	-1.651282	-1.504447
C	-0.000000	-3.016477	-1.211823
C	-0.000000	-3.461910	0.114800
C	-0.000000	-2.545382	1.172195
H	-0.879243	0.000000	2.492131
H	0.879243	-0.000000	2.492131
H	0.000000	2.897360	2.201386
H	0.000000	4.528206	0.324971
H	0.000000	3.740970	-2.022058
H	0.000000	1.311700	-2.537216
H	-0.000000	-1.311700	-2.537216
H	-0.000000	-3.740970	-2.022058
H	-0.000000	-4.528206	0.324971
H	-0.000000	-2.897360	2.201386

anthracene

C	3.660757	0.713148	-0.000020
C	2.479588	1.407013	0.000048
C	1.223955	0.722629	0.000033
C	1.223957	-0.722629	0.000024
C	2.479590	-1.407012	-0.000026
C	3.660758	-0.713144	-0.000065
C	-0.000000	1.403347	0.000027
C	0.000001	-1.403348	0.000031
C	-1.223955	-0.722631	0.000027
C	-1.223956	0.722628	0.000008
C	-2.479589	1.407013	-0.000028
H	-2.476995	2.494558	-0.000071
C	-3.660758	0.713147	-0.000059
C	-3.660757	-0.713146	-0.000009
C	-2.479589	-1.407013	0.000030
H	-0.000006	2.491724	0.000048
H	4.607430	1.246662	-0.000048
H	2.476992	2.494558	0.000099
H	2.476996	-2.494558	-0.000029
H	4.607426	-1.246667	-0.000125
H	-0.000005	-2.491726	0.000048
H	-4.607427	1.246666	-0.000096
H	-4.607427	-1.246665	-0.000027
H	-2.476999	-2.494559	0.000076

resveratrol

C	-3.647795	1.246459	0.000818

C	-2.266366	1.077492	0.001108
C	-1.715926	-0.216961	0.000317
C	-2.579608	-1.325329	-0.000613
C	-3.962444	-1.143105	-0.000891
C	-4.510397	0.142407	-0.000200
H	-1.643237	1.964191	0.002126
H	-2.186688	-2.336962	-0.001214
H	-5.590825	0.284522	-0.000376
C	-0.271278	-0.470938	0.000450
H	-0.004390	-1.526407	0.001094
C	0.712932	0.451402	-0.000328
H	0.438332	1.505354	-0.001359
C	2.156329	0.210017	-0.000114
C	3.031368	1.310599	-0.002253
C	2.738892	-1.075232	0.002169
C	4.414462	1.149852	-0.002241
H	2.617084	2.316095	-0.004002
C	4.114482	-1.250590	0.002205
H	2.104092	-1.956200	0.004037
C	4.963475	-0.135253	-0.000024
H	5.065820	2.022265	-0.003947
H	4.554333	-2.243012	0.003991
O	6.309916	-0.369118	0.000142
H	6.777215	0.480825	-0.001506
O	-4.742778	-2.267381	-0.001861
H	-5.676022	-2.004854	-0.001993
O	-4.121381	2.531231	0.001630
H	-5.090526	2.511406	0.001440

chlorophyll

MG	-6.037602	0.492182	0.250177
C	-3.377298	-1.582397	-0.494452
C	-4.101018	3.192546	-0.536643
C	-8.684793	2.503584	0.936046
C	-7.946732	-2.309840	1.050083
N	-4.026456	0.745018	-0.448925
C	-3.119846	-0.248249	-0.738976
C	-1.883050	0.327751	-1.405765
C	-1.984346	1.826034	-1.022365
C	-3.465300	1.964107	-0.666401
C	-1.087923	2.208067	0.169779
C	-1.940320	0.100835	-2.941423
C	-0.558504	0.175758	-3.611670
C	0.229718	-1.097279	-3.339962
O	-0.210503	-2.213627	-3.532570
O	1.460523	-0.842785	-2.867792
N	-6.341005	2.500192	0.198519
C	-5.433239	3.456529	-0.150027
C	-6.037140	4.773389	-0.059420
C	-7.340031	4.581555	0.362854
C	-7.521037	3.140706	0.515634
C	-5.359311	6.061263	-0.415519
C	-8.392338	5.566357	0.590865
C	-8.234713	6.842406	0.975651
N	-7.978658	0.151266	0.886735
C	-8.912646	1.121523	1.111566
C	-10.157169	0.523945	1.553433
C	-9.948783	-0.838407	1.586516
C	-8.576216	-1.058579	1.166572
C	-11.403771	1.282382	1.902359

C	-10.914147	-1.910646	2.011010
C	-10.765516	-2.317878	3.490015
N	-5.761281	-1.506936	0.326661
C	-6.627749	-2.548671	0.652704
C	-5.952594	-3.825582	0.495772
C	-4.669119	-3.501207	0.071796
C	-4.607111	-2.084936	-0.016053
C	-6.527282	-5.187423	0.734770
C	-3.377061	-4.047855	-0.346734
O	-3.010243	-5.202672	-0.451935
C	-2.457058	-2.799803	-0.675868
C	-1.264598	-2.728747	0.271856
O	-0.138428	-2.395996	-0.039200
O	-1.619880	-3.036163	1.534869
C	-0.568323	-2.973468	2.511621
C	2.254785	-2.020676	-2.523940
C	3.550394	-1.533463	-1.960342
C	4.793102	-1.839971	-2.364948
C	5.120497	-2.763113	-3.514498
C	5.990197	-1.255877	-1.638092
C	6.732131	-0.158967	-2.430319
C	7.933910	0.409783	-1.661797
C	8.660777	1.573285	-2.368947
C	9.358005	1.128330	-3.665806
C	9.627494	2.316320	-1.417124
C	10.803922	1.499314	-0.858216
C	11.702257	2.343578	0.060527
C	12.928671	1.631488	0.679830
C	13.919603	1.150169	-0.393138
C	12.508052	0.498329	1.640766
C	13.631721	-0.141703	2.479064
C	14.310625	0.822793	3.464782
C	15.358706	0.199679	4.416617
C	16.556949	-0.399709	3.663632
C	14.747546	-0.824222	5.386224
H	-3.486906	4.061481	-0.752931
H	-9.525154	3.149176	1.170647
H	-8.549561	-3.181087	1.289706
H	-0.972372	-0.133851	-1.009487
H	-1.729772	2.470833	-1.872369
H	-1.239879	3.253068	0.460649
H	-0.030896	2.072406	-0.086025
H	-1.308824	1.581216	1.041521
H	-2.614501	0.842751	-3.386813
H	-2.363681	-0.883268	-3.163840
H	0.011816	1.051059	-3.286286
H	-0.685292	0.247038	-4.699282
H	-4.622332	5.919396	-1.213125
H	-4.829234	6.502429	0.440244
H	-6.088770	6.801622	-0.759820
H	-9.410363	5.210644	0.433646
H	-9.093796	7.494613	1.103436
H	-7.263133	7.270948	1.199623
H	-12.243538	0.607019	2.091178
H	-11.704644	1.962041	1.095143
H	-11.269253	1.894700	2.804005
H	-10.786261	-2.798664	1.378662
H	-11.941466	-1.568423	1.836809
H	-10.941268	-1.461681	4.150434

H	-9.756934	-2.692450	3.696324
H	-11.482437	-3.104534	3.752571
H	-7.403000	-5.373786	0.099964
H	-6.852321	-5.311785	1.775869
H	-5.779998	-5.954779	0.517690
H	-2.045920	-2.898029	-1.686076
H	-1.029539	-3.266737	3.454846
H	-0.166712	-1.958582	2.578215
H	0.239508	-3.660665	2.247751
H	2.364586	-2.636981	-3.418261
H	1.678364	-2.589390	-1.787495
H	3.436786	-0.870012	-1.102888
H	4.240170	-3.092615	-4.070515
H	5.638048	-3.660682	-3.148360
H	5.802068	-2.280055	-4.225692
H	6.702959	-2.061170	-1.402205
H	5.665419	-0.837465	-0.677139
H	6.024965	0.650137	-2.660437
H	7.064491	-0.562267	-3.394820
H	8.646317	-0.403142	-1.459762
H	7.585707	0.759752	-0.678406
H	7.890422	2.308415	-2.650457
H	8.639508	0.753795	-4.402760
H	10.085051	0.328457	-3.479783
H	9.895391	1.965052	-4.128563
H	9.045169	2.715569	-0.573390
H	10.028821	3.192624	-1.947566
H	11.400209	1.097399	-1.687154
H	10.419447	0.633266	-0.305024
H	11.087262	2.748992	0.877798
H	12.060700	3.215237	-0.507119
H	13.443584	2.399179	1.275552
H	14.862973	0.813886	0.050731
H	14.158249	1.956925	-1.097020
H	13.513825	0.311966	-0.972671
H	12.019424	-0.297330	1.061446
H	11.741167	0.889399	2.326440
H	14.381881	-0.584891	1.811843
H	13.193532	-0.980653	3.035485
H	13.532522	1.305990	4.074896
H	14.800799	1.631202	2.904967
H	15.743524	1.030636	5.027046
H	17.004738	0.331210	2.978950
H	16.265960	-1.276377	3.072549
H	17.336869	-0.722283	4.363753
H	13.898929	-0.397353	5.934790
H	15.489602	-1.153913	6.123245
H	14.390366	-1.718484	4.861693
